# Hexavalent chromium removal and total chromium biosorption from aqueous solution by *Quercus crassipes* acorn shell in a continuous up-flow fixed-bed column: Influencing parameters, kinetics, and mechanism

**DOI:** 10.1371/journal.pone.0227953

**Published:** 2020-01-21

**Authors:** Erick Aranda-García, Eliseo Cristiani-Urbina

**Affiliations:** Instituto Politécnico Nacional, Escuela Nacional de Ciencias Biológicas, Departamento de Ingeniería Bioquímica, Avenida Wilfrido Massieu s/n, Unidad Profesional Adolfo López Mateos, Delegación Gustavo A. Madero, Ciudad de México, México; King Saud University, SAUDI ARABIA

## Abstract

Continuous fixed-bed column studies were carried out, utilizing acorn shell from *Quercus crassipes* Humb. & Bonpl. (QCS), in order to remove total chromium and Cr(VI) from aqueous solution. Effects of various fixed-bed column parameters such as influent solution pH, influent flow rate, QCS bed height, and influent Cr(VI) concentration were investigated. Results from the fixed-bed column experiments demonstrate that total chromium biosorption and Cr(VI) removal by QCS depend strongly on the pH of influent solution. The highest capacities for Cr(VI) removal and total chromium biosorption are about 181.56 and 110.35 mg g^-1^ and are achieved at influent solution pH of 1.0 and 2.0, respectively. Besides this, total chromium biosorption capacities increased from 104.25 to 116.14 mg g^-1^, 109.07 to 117.44 mg g^-1^, and 85.02 to 129.87 mg g^-1^, as bed height, inlet flow rate, and influent Cr(VI) concentration increased from 1.7 to 6.5 cm, 0.25 to 1 mL min^-1^, and 50 to 400 mg L^-1^, respectively. The dose–response model defines the entire breakthrough curve for total chromium biosorption onto QCS, under all experimental conditions. X-ray photoelectron spectroscopy (XPS) and biosorption kinetic studies revealed that QCS is able to remove toxic Cr(VI) from acidic liquid solution by means of a complex mechanism that involves the binding of Cr(VI) oxyanions to positively charged groups present at the QCS surface, after which the Cr(VI) species are reduced to Cr(III) by adjacent electron donor groups, and the generated Cr(III) ions then become partially bound to the QCS biomass and partially released into the liquid phase. Results show that QCS can be employed as an easily accessible, abundant, eco-friendly, and inexpensive biosorbent for the removal of total chromium and Cr(VI) from Cr(VI) solutions, in continuous operation.

## Introduction

Chromium [Cr] compounds are among the most common environmental contaminants because of their widespread and extensive use in industrial applications [1]. The trivalent [Cr(III)] and hexavalent [Cr(VI)] oxidation states of chromium are the most stable and abundant in the natural environment; they differ significantly, however, in their physicochemical properties, bioavailability, and toxicity to living organisms [2].

Hexavalent chromium [Cr(VI)] is one of the most poisonous and harmful heavy metals, one of the top 20 pollutants on the Superfund priority list of hazardous substances [3], the third most common pollutant at hazardous waste sites [4], and the number one carcinogenic substance [5]. In addition, Cr(VI) is highly water-soluble, highly mobile in aqueous systems, and highly toxic as it causes severe physiological and neurological ill effects on human and animal health, such as anemia; diarrhea; nausea; epigastric pain; ulcers; vomiting; eye and skin irritation; damage to nerve tissues, kidney, and liver; pulmonary congestion; internal hemorrhaging; and circulatory shutdown [1, 6]. It is also highly mutagenic and can induce DNA damage and birth defects, affect gene expression, and decrease reproductive health [1, 7]. Furthermore, Cr(VI) is toxic to plants as it severely affects several of their biochemical, physiological, and morphological processes, leading to slow growth, chlorosis, and necrosis [1]. Contrastingly, Cr(III) is relatively immobile in the environment owing to its low solubility in water. At low concentrations, Cr(III) is an essential micronutrient for animals and humans as it plays a significant role in the normal metabolism of carbohydrates, lipids, and proteins [1, 2, 6]; at high concentrations, however, Cr(III) has adverse effects on cellular structure and function [7].

Several conventional physical, chemical, and biological methods have been developed to remediate Cr(VI)-contaminated wastewater and water, and thereby avoid or reduce the adverse effects of highly toxic Cr(VI) on human health and the environment [1, 7]. However, incomplete metal removal processes, ineffectiveness at low metal concentrations, high energy inputs and costs, requirement for high amounts of chemical agents, and generation of toxic by-products as well as difficulties and costs in managing and treating them, limit the use of conventional methods in larger applications [6, 8, 9]. It is thus crucial to develop novel, effective and economic remediation technologies for removing Cr(VI) from Cr(VI)-bearing aqueous solutions [10].

Biosorption has been seen as a promising and attractive technology for removing Cr(VI) from polluted industrial wastewaters and aqueous environments in an effective, competitive, economical, and eco-friendly manner [1, 11]. However, the complex nature of the Cr(VI) biosorption removal from acidic solutions, which involves a complex mechanism of biosorption and biotransformation of the mobile and highly toxic Cr(VI) to the immobile and less toxic Cr(III) [2, 7, 12–15], makes this task particularly challenging.

To date, most of the research on the biosorptive removal of Cr(VI) has been conducted in batch systems, which are very easy to apply at the laboratory scale but difficult to use on a large scale, particularly when the volume of the industrial effluent requiring treatment is large [16, 17]. In addition, the data on batch systems may not be applicable to most treatment systems such as continuous fixed-bed column operations, in which contact time is not sufficiently long for attainment of equilibrium [18]. Therefore, it is crucial to ascertain the practical applicability of a biosorbent in the continuous mode [16].

In the practical operation of a large-scale biosorption process, fixed-bed column systems are preferred for continuous wastewater treatment and cyclic biosorption/desorption of heavy metals because of their high effectiveness, high biosorption/desorption capacity, simplicity of operation, low cost, and capacity to be scaled up from a laboratory process and produce effluents of higher quality [16, 19, 20].

Few studies exist regarding the dynamic performance of continuous fixed-bed biosorption columns for chromium removal from Cr(VI) aqueous solutions. Moreover, the authors of those studies only measured either the effluent concentration of Cr(VI) or that of total chromium, but not that of both concentrations; meaning they did not assess the biotransformation of Cr(VI) to Cr(III), caused by the biosorbents being assayed. Likewise, several kinetic evaluations were made without taking into account the Cr(VI) biotransformation process, thus leading to erroneous conclusions. Therefore, there are no reports in the specialized literature that consider the effect of influencing parameters for the removal of both Cr(VI) and total chromium from Cr(VI) aqueous solutions, in continuous fixed-bed columns.

Furthermore, solution pH is considered the most important experimental parameter that controls the biosorptive removal of chromium ions from aqueous solution because of its strong influence on the degree of dissociation of functional groups from the biosorbent surface as well as on the charge, chemical speciation, and solubility of the chromium compound in aqueous solution and on the competition with coexisting ions in solution [2, 14]. However, in spite of its great importance and relevance for the processes of biotransformation of Cr(VI) to Cr(III) and chromium biosorption, most researchers have not investigated the effect of influent solution pH on the removal of both total chromium and Cr(VI), in continuous fixed-bed column systems. Apparently, it has been assumed that the optimum solution pH for chromium biosorption, determined in batch system will mirror that of the continuous system. However, the operating conditions of batch and continuous biosorption systems are very different; it is thus reasonable to suppose that the optimum pH for the biosorption of a target pollutant may differ in the two systems. This is especially true in the case of redox-sensitive heavy metals such as chromium, because their charge and chemical speciation are highly dependent on solution pH [2, 17].

As far as we know, only three previous works have addressed the effect of influent Cr(VI) solution pH on chromium removal in continuous fixed-bed column systems [2, 21, 22]. Nevertheless, in two of those three studies only Cr(VI) was measured (not total chromium), and consequently the authors of those two studies did not realize the occurrence of biotransformation of Cr(VI) to Cr(III) by the biosorbents assayed, which led to incorrect conclusions [21, 22]. In contrast, Aranda-García and Cristiani-Urbina [2] measured both total chromium and Cr(VI) concentrations throughout the continuous fixed-bed column experiments and demonstrated that influent solution pH causes differences in performance in the removal of total chromium and Cr(VI) and strongly affects the biotransformation of Cr(VI) to Cr(III) and biosorption of chromium ions. There is therefore a clear need to investigate the influence of inlet solution pH on the removal of both total chromium and Cr(VI), in continuous fixed-bed column systems.

Acorn shell from *Quercus crassipes* Humb. & Bonpl. was previously reported as one of the best biosorbents currently available for the biosorption of total chromium from Cr(VI) aqueous solutions in batch systems [12, 23], and it is therefore of great scientific and technological interest to study its capacity performance for continuous fixed-bed column biosorption, under different operating conditions. To the best of our knowledge, the use of QCS in a continuous-flow system has not yet been reported.

The present study focused on exploring the practical applicability of QCS as a biosorbent in a continuous fixed-bed up-flow column for the removal of total chromium and Cr(VI) from aqueous solution. The effects of relevant operational parameters, such as the pH, flow rate, and Cr(VI) concentration in the influent Cr(VI) solution, as well as the QCS bed height on the removal of total chromium and Cr(VI) were investigated. The experimentally obtained results were modeled by the application of the Thomas, Bohart–Adams, Yan, dose-response, and Yoon–Nelson models, and the models' parameters were determined. Furthermore, the Cr(VI) removal mechanism from aqueous solution by QCS was elucidated by monitoring the kinetic changes of total chromium and Cr(VI) concentrations, and using X-ray photoelectron spectroscopy (XPS).

## Material and methods

### Ethics statement

No specific permits were required for the acorn sample collection for this study. We used acorns from the *Quercus crassipes* Humb. & Bonpl. tree, collected from the private land belonging to one of the authors (EAG). No acorn material was removed from protected land or National Parks. The studies conducted in this work did not involve any protected or endangered species.

### Cr(VI) stock solution

A Cr(VI) stock solution of 1 g L^-1^ was prepared by dissolving 3.74 g of K_2_CrO_4_ (99.9% purity; J.T. Baker®, Mexico) in 1 liter of distilled deionized water. Test solutions of Cr(VI) were prepared by diluting the stock solution with distilled deionized water, and the pH of each solution was adjusted to the desired value using 0.1 M HCl or NaOH (J.T. Baker®, Mexico) solutions.

### Experimental flow sheet

**Fig 1:**
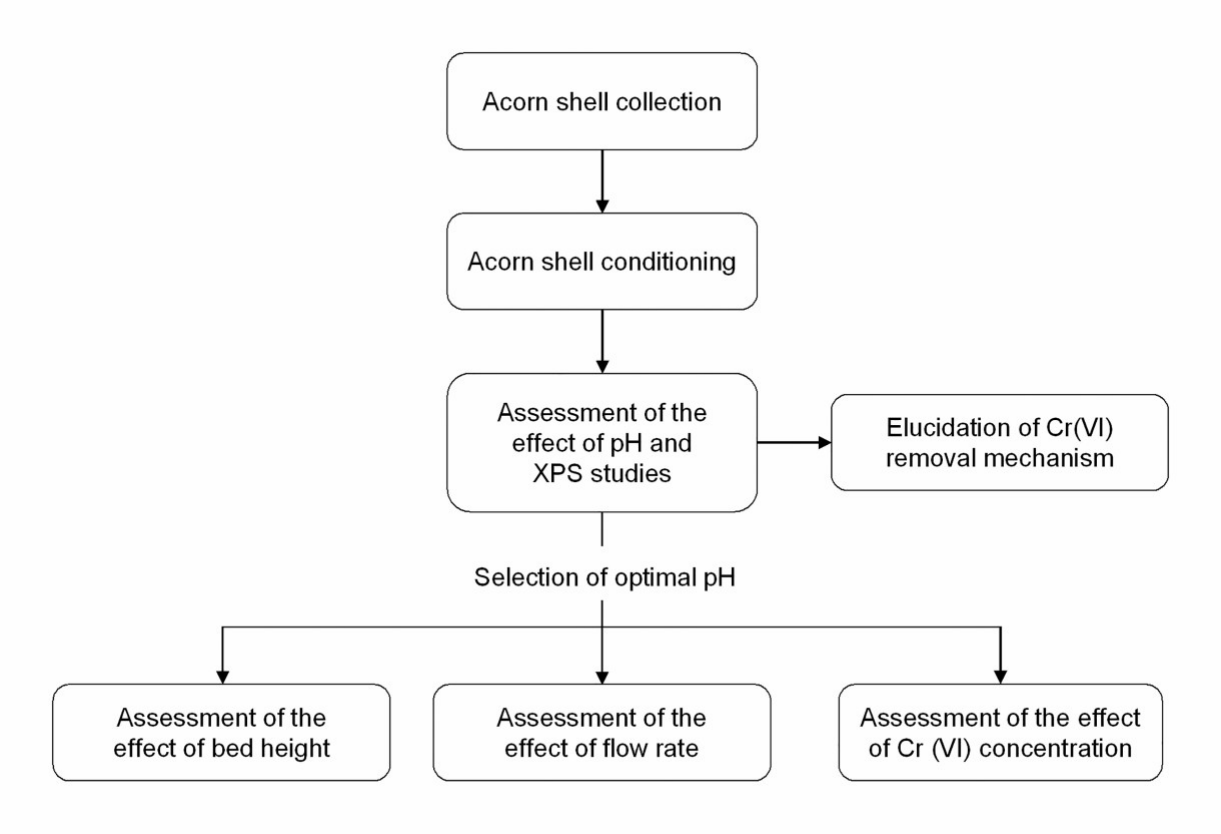
Schematic flow sheet of the experimental setup.

Fig 1 provides a schematic flow sheet of the experimental setup, and the stages of the process are described in detail below.

### Biosorbent material

Acorn samples from the *Quercus crassipes* Humb. & Bonpl. tree were collected in the locality of El Durazno (19.916017, -99.610222), town of Jilotepec, Mexico State, Mexico. The acorns were washed thoroughly with running water followed by distilled deionized water. Subsequently, they were dried at 60°C until the dry weight was constant. Afterward, the shells were separated from the acorns and ground in a laboratory scale hammer mill (Glen Creston®), and the resulting particles were sieved through standard ASTM testing sieves. The fraction with particle sizes of 150–500 μm was used. The sieved biosorbent, without any further chemical or physical treatment, was stored in a closed plastic container until ready for use in the total chromium and Cr(VI) removal experiments. The mean size of the QCS particles was 445.1 μm, determined using a Beckman Coulter LS 13320 particle size analyzer.

The QCS proximate chemical composition was analyzed in triplicate following the methods outlined by the Association of Official Analytical Chemists [24], and the results were as follows: crude fiber, 50.72%; ether extract, 0.49%; nitrogen-free extract, 45.79%; total ash, 1.12%; and total protein, 1.88%, which indicate that QCS is composed of a low content of proteins, lipids, and ashes and a high content of carbohydrates.

The QCS's point of zero charge (pH_PZC_) was found to be 5.4 as determined using a batch equilibrium method and following the procedures described by Hasan et al. [25]. The density of QCS particles was 1.0017 g cm^−3^.

Furthermore, Fourier transform infrared (FTIR) spectroscopic analysis of raw QCS was conducted in a Perkin-Elmer Spectrum 2000 FTIR spectrophotometer, equipped with a Perkin-Elmer diffuse reflectance FTIR accessory. The FTIR spectrum for raw QCS was reported previously [26] revealing a large number of absorption peaks within an interval of 4000 to 400 cm^-1^ that reflect the complex chemical nature of QCS. Absorption bands, corresponding to aromatic compounds such as lignin and tannins, cellulose molecules, O-H groups, alkyl radicals and other saturated aliphatic groups, as well as carboxyl groups were detected.

### Removal of total chromium and Cr(VI) in a continuous up-flow fixed-bed reactor

Fixed-bed column biosorption studies were performed to evaluate dynamic behavior for the removal of total chromium and Cr(VI) by QCS. The fixed-bed column system used in the present work was previously tested and described by Aranda-García and Cristiani-Urbina [2]. The continuous biosorption system consisted of a feed tank, a peristaltic feed pump, a fixed-bed column with inlet and outlet ports, and a fraction collector. A Pyrex glass cylindrical column with an internal diameter of 1.2 cm and a height of 15.5 cm was employed in the column experiments. It is well known that wall effects can influence the shape of the biosorption breakthrough curves for fixed-bed systems and that for avoiding such effects it is recommended that the ratio of column diameter (*D*_C_) to particle diameter (*D*_p_) be greater than 10 [20]. The present study complied with this recommendation. The column was packed with the various desired quantities of QCS, and the bulk density and porosity of the fixed bed were 0.5556 g cm^−3^ and 0.56, respectively. The QCS bed was packed between layers of 3-millimeter-diameter spherical glass beads, with a height of glass beads of 6 cm and 3.5 cm at the bottom and top of the fixed bed, respectively. The glass beads in the lower portion of the column were used in order to support the QCS bed, to ensure uniform distribution of the influent across the entire cross section of the column, and to dampen the fluctuating flow, a phenomenon induced by the peristaltic pump [2]. The top portion of glass beads was used to avoid the washing out of QCS particles. In addition, a 300-mesh sieve was placed between the QCS biomass and the glass beads to prevent any loss of biomaterial. The column was joined to the lid and base by stainless steel clamps and was sealed with silicone rubber gaskets. At the center of the reactor base, there was a liquid diffuser with a pore size of 30–40 μm.

The experiments were performed by pumping Cr(VI) solution at a desired flow rate in an upward-flow mode through the biosorbent bed using a peristaltic pump (Masterflex L/S 07528-30) at room temperature (20 ± 2°C). The influence of influent pH on total chromium biosorption and Cr(VI) removal in the fixed-bed column was investigated at five different pH values, namely 1.0, 1.5, 2.0, 2.5, and 3.0, and using an inlet Cr(VI) concentration, Cr(VI) solution flow rate, QCS bed height, and total operation time of 200 mg L^−1^, 0.75 mL min^−1^ (equivalent to a volumetric flux of 398.23 L m^−2^ h^−1^), 3.5 cm (equivalent to a QCS bed mass of 2 g), and 96 h, respectively. The influent solution pH at which the highest total chromium removal was achieved was selected as the optimum pH and used in further studies.

In order to examine the effect of bed height, fixed-bed studies were performed with bed heights of 1.7, 3.5, 5.0, and 6.5 cm, with biosorbent dosages of 1, 2, 3, and 4 g of QCS, respectively, using a Cr(VI) solution flow rate of 0.75 mL min^−1^ (398.23 L m^−2^ h^−1^), an inlet Cr(VI) concentration of 200 mg L^−1^, and a fixed-bed column operation time of 144 h. When the influence of Cr(VI) solution flow rate was studied, the bed height was 3.5 cm (2 g of QCS), the inlet Cr(VI) concentration was 200 mg L^−1^, the total operation time of the fixed-bed column was 144 h, and the flow rates were 0.25, 0.5, 0.75, and 1.0 mL min^−1^, which correspond to Cr(VI) solution volumetric fluxes of 132.74, 265.49, 398.23, and 530.97 L m^−2^ h^−1^, respectively. Furthermore, the biosorption performance of QCS fixed bed was tested at four influent Cr(VI) concentrations, namely 50, 100, 200, and 400 mg L^−1^, at a Cr(VI) solution flow rate of 0.75 mL min^−1^ (398.23 L m^−2^ h^−1^), a bed height of 3.5 cm (2 g), and a fixed-bed column operation time of 144 h.

Liquid effluent samples were collected intermittently at the exit of the fixed-bed column using a programmable fraction collector (LKB Bromma, Ltd.), filtered through Whatman 42 ashless filter paper, and the biosorbent-free filtrates were subsequently analyzed spectrophotometrically for residual total chromium and Cr(VI) concentrations.

QCS-free controls were used to assess the loss of chromium ions arising from precipitation and/or adsorption on glass wall. No measurable changes in the influent and effluent total chromium and Cr(VI) concentrations were detected in any of the assayed QCS-free controls, thus confirming that the observed decrease in total chromium and Cr(VI) concentrations in the experiments using QCS was due only to the QCS biosorbent.

### Fixed-bed column performance evaluation

To evaluate the fixed-bed column performance, the breakthrough curves were obtained by plotting the ratio between total chromium or Cr(VI) concentrations at the outlet (*C_t_*, mg L^−1^) and inlet (*C*_0_, mg L^−1^) of the column as a function of operation time (*t*, h). The mass (*m*_r_, g) of total chromium or Cr(VI) removed by the fixed-bed column was estimated as follows [2]:

$$m_{r}=\frac{Q}{1000}\int_{t=0}^{t=t_{T}}(1-\frac{C_{t}}{C_{0}})C_{0}\;dt$$ (1)

Here *Q* is the flow rate of the influent Cr(VI) solution (mL min^−1^), and *t*_T_ is the total operation time of the fixed-bed column (h).

Likewise, the total chromium or Cr(VI) removal capacity (*q*_b_, mg g^−1^) was determined using the following equation [2]:

$$q_{b}=\frac{m_{r}}{m_{b}}$$ (2)

Here, *m*_b_ is the mass of the QCS biomass packed in the column (g).

Additionally, the maximum volumetric biosorption capacity (*N*_b_, mg L^−1^) was calculated considering the fixed-bed bulk density (*ρ*_b_, g L^−1^), as follows:

$$N_{b}=q_{b}\rho_{b}$$ (3)

### Mathematical modeling of total chromium biosorption breakthrough curves

Modeling the biosorption breakthrough curve is crucial for deriving characteristic or design parameters that can be used to describe the breakthrough curve for given experimental conditions as well as to predict the breakthrough curve behavior for scale-up purposes and performance comparisons [20, 27]. In this work, the Thomas, Bohart–Adams, Yan, dose–response and Yoon–Nelson models were fitted to the data from the dynamic flow experiments performed in the fixed-bed column to predict the breakthrough curves obtained at different influent Cr(VI) solution pH values, influent Cr(VI) concentrations, biosorbent bed heights, and influent flow rates as well as to determine the characteristic parameters of the column.

The Bohart–Adams model can be written using Eq 4 [28]:

$$\frac{C_{t}}{C_{0}}=\frac{e^{k_{AB}C_{0}t}}{e^{k_{AB}N_{0}Z/v}-1+e^{k_{AB}C_{0}t}}$$ (4)

Here *C_t_* is the total chromium concentration of the outlet effluent (mg L^−1^) at time *t* (h), *C*_0_ is the influent total chromium concentration (mg L^−1^), *k*_AB_ is the kinetics constant of the Bohart–Adams model (L^−1^ mg^−1^ min^−1^), *N*_0_ is the maximum volumetric biosorption capacity (mg L^−1^), *Z* is the biosorbent bed height in the column (cm), and *v* is the linear flow rate (cm min^−1^).

The Thomas model is given as follows [28]:

$$\frac{C_{t}}{C_{0}}=\frac{1}{1+e^{\left(\frac{k_{Th}}{Q})({q_{Th}m_{b}-C}_{0}Qt\right)}}$$ (5)

Here *k*_Th_ is the rate constant of the Thomas model (mL min^−1^ mg^−1^), *Q* is the influent Cr(VI) solution flow rate (mL min^−1^), *q*_Th_ is the maximum total chromium concentration in the biosorbent (mg g^−1^), and *m*_b_ is the total mass of biosorbent in the column (g).

The model of Yoon–Nelson has the following form [18]:

$$\frac{C_{t}}{C_{0}}=\frac{e^{k_{YN}(t-\tau )}}{1+e^{k_{YN}(t-\tau )}}$$ (6)

Here *k*_YN_ is the Yoon–Nelson model rate constant (min^−1^), and *τ* is the operation time (min) at which *C_t_*/*C*_0_ is equal to 0.5.

The Yan model can be written using Eq 7 [2]:

$$\frac{C_{t}}{C_{0}}=1-\frac{1}{1+({\frac{C_{0}\;Q\;t}{q_{0}m_{b}})}^{a}}$$ (7)

Here *a* is the constant of the Yan model (dimensionless), and *q*_0_ is the maximum capacity of biosorption (mg g^−1^).

The dose–response model has the following form [27]:

$$\frac{C_{t}}{C_{0}}=1-\frac{1}{1+({\frac{t}{\beta })}^{\alpha }}$$ (8)

Here *α* and *β* are the constants of the dose–response model. *β* is the operation time (min) at which *C_t_*/*C*_0_ is equal to 0.5.

### Statistical analysis and data analysis

Data on the removal of total chromium and Cr(VI) were statistically analyzed by ANOVA with Tukey's test, and with a significance level of *p* < 0.05, using GraphPad Prism software version 7.0 (GraphPad Software, Inc., La Jolla, CA, USA). The same software was used to obtain all the kinetic parameters for the fixed-bed models by nonlinear regression methods. The best-fit model was selected by the highest determination coefficient (*r*^2^), the lowest sum of squared errors (SSE), root mean squared error (RMSE), Akaike information criterion (AIC), and the narrowest 95% confidence intervals.

### Analytical methods

#### Quantification of total chromium and Cr(VI) concentrations

Total chromium (essentially, the sum of the Cr(III) and Cr(VI)) and Cr(VI) concentrations in influent and effluent solutions were quantified colorimetrically using a Thermo Scientific^™^ Evolution 201 spectrophotometer according to the procedures described in Hach methods 8024 and 8023 of the Hach *Water Analysis Handbook* [29], respectively. Cr(III) concentration in solution was calculated by subtracting residual Cr(VI) concentration from residual total chromium concentration [29]. Total chromium and Cr(VI) concentrations are directly proportional to their absorbance and were assessed by external standards using a calibration curve.

#### X-ray photoelectron spectroscopy analysis

Chromium-unloaded (raw biosorbent) and chromium-loaded QCS samples were assessed using X-ray photoelectron spectroscopy (XPS) to detect chromium ions at the QCS surface, to determine the redox state of the chromium biosorbed onto QCS, and to elucidate the mechanism involved in chromium binding to QCS.

The chromium-loaded QCS biomass was obtained from the continuous fixed-bed column experiments performed at the various influent Cr(VI) solution pH levels (1.0–3.0); this was done because the solution pH is the main environmental parameter that affects the oxidation–reduction state of chromium. Afterward, the chromium-loaded QCS samples were washed thoroughly with distilled deionized water, dried at 60°C until the dried weight was constant, and then stored in a closed plastic container until XPS analysis.

XPS spectra were recorded on a Thermo Fisher Scientific K-Alpha spectrometer by applying an Al Kα (1486.6 eV) energy source of focused monochromatic radiation. The system was operated at a base pressure of 10^−9^ mbar. Low-resolution survey spectra were obtained using a pass energy of 200 eV over a binding energy range of −10 to 1370 eV, whereas Cr 2p high-resolution spectra were recorded with a pass energy of 40 eV over a binding energy range of 568 to 597 eV. The analytical grade compounds K_2_CrO_4_ and K_2_Cr_2_O_7_ were employed as Cr(VI) references; likewise, CrCl_3_·6H_2_O, Cr(NO_3_)_3_, and Cr_2_O_3_ (J.T. Baker®, Mexico) were employed as Cr(III) references. The existence of chromium ions on the QCS surface was determined by comparing XPS spectra of raw and chromium-loaded QCS samples. The comparison and analysis of experimental XPS spectra with those of Cr(VI) and Cr(III) reference compounds were performed to identify the oxidation state of the chromium biosorbed onto QCS.

## Results and discussion

### Effect of feed Cr(VI) solution pH on removal of total chromium and Cr(VI)

**Fig 2:**
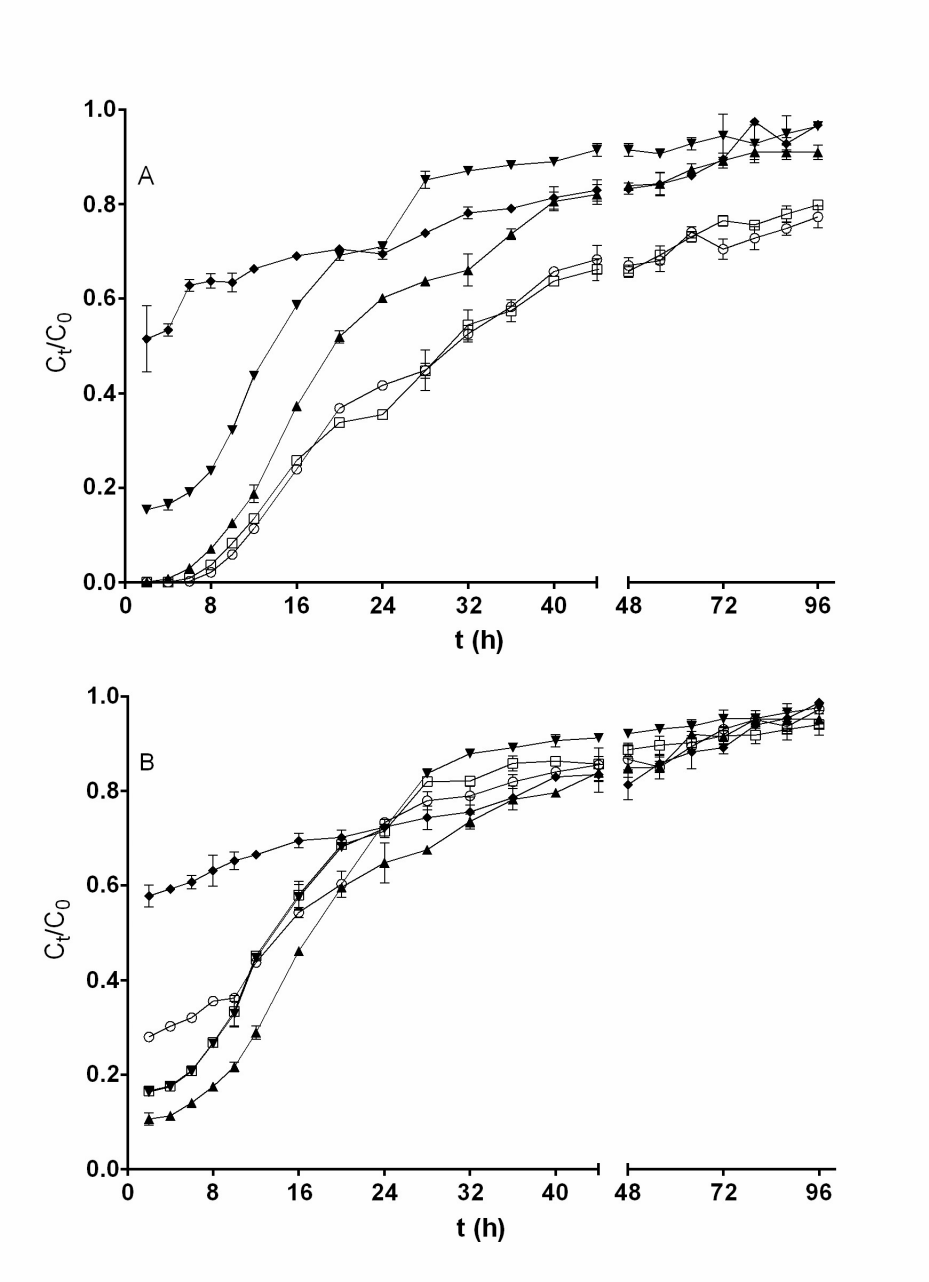
**Influence of influent pH on the breakthrough curve for removal of Cr(VI) (A) and total chromium (B) by QCS [influent pH: ○, 1.0; □, 1.5; ▲, 2.0; ▼, 2.5; ◆, 3.0].** Conditions: influent Cr(VI) concentration, 200 mg L^−1^; influent flow rate, 0.75 mL min^−1^; bed height, 3.5 cm. When they do not appear, standard deviation bars are smaller than the size of the symbols.

Fig 2 shows breakthrough curves for the removal of total chromium and Cr(VI) at various influent Cr(VI) solution pH values ranging from 1.0 to 3.0. It is evident that effluent total chromium and Cr(VI) concentrations varied widely with the influent Cr(VI) solution pH. The lowest concentrations of total chromium and Cr(VI) in the effluents were attained in the first hours of operation of the fixed-bed column; thereafter, the effluent concentrations increased progressively as the operation time increased, indicating a decrease in the removal capacities for total chromium and Cr(VI) due to the gradual exhaustion of active sites for biosorption of chromium ions and bioreduction of Cr(VI) [30].

No measurable concentration of Cr(VI) was found in the outlet effluent at column operation times less than 6 h with pH values of the influent Cr(VI) solution of 1.0, 1.5, or 2.0. Contrastingly, when the influent pH was 2.5 or 3.0, Cr(VI) was present in the effluent from the beginning of the column operation (Fig 2A). Throughout the column operation period, the lowest levels of Cr(VI) in the effluent were achieved at influent pH values of 1.0 and 1.5.

Furthermore, it was noted that with a decrease in the pH of the influent solution, the breakthrough curves for Cr(VI) removal shifted from left to right, which indicates that more Cr(VI) ions were removed by the fixed QCS bed. Consequently, the highest removal capacity of Cr(VI) was observed at an influent pH of 1.0, and as the solution pH increased from 1.0 to 3.0, the removal capacity decreased from 181.56 to 75.18 mg g^−1^ (Table 1). The highest capacities for the removal of Cr(VI) of beds packed with Hass avocado (*Persea americana* Mill var. Hass) shell [2] and *Quercus ithaburensis* acorn [22] were achieved at influent pH values of 1.0–1.5 and 2.0, respectively, which were the lowest pH values for which assays were performed in those studies. The Cr(VI) removal capacity shown by QCS in the fixed-bed column was similar to that of Hass avocado shell and markedly greater than that of *Quercus ithaburensis* at all influent pH values for which assays were performed.

**Table 1:** Parameters of breakthrough curves for removal of total chromium and Cr(VI) by QCS in fixed-bed columns under different operational conditions.

							Cr(VI)		Total chromium	
pH	*t_T_ (h)*	*H (cm)*	*Q (mL min^-1^)*	*C_0_ (mg L^-1^)*	*N_b_ (mg/L)*	*t_50_ (min)*	*m_r_ (mg)*	*q_b_* (mg g^-1^)	*m_r_ (mg)*	*q_b_* (mg g^-1^)
1.0	96	3.5	0.75	200	48198 ± 617.0	797.16 ± 45.13	363.11 ± 0.84	181.56 ± 0.42	173.50 ± 2.22	86.75 ± 1.11
1.5	96	3.5	0.75	200	49821 ± 1961	785.33 ± 40.07	352.30 ± 6.42	176.15 ± 3.21	179.35 ± 7.06	89.67 ± 3.53
2.0	96	3.5	0.75	200	61310 ± 339.0	1072.6 ± 38.65	246.67 ± 3.55	123.34 ± 1.77	220.70 ± 1.23	110.35 ± 0.61
2.5	96	3.5	0.75	200	41831 ± 111.0	785.77 ± 40.04	160.64 ± 3.09	80.31 ± 1.55	150.59 ± 0.41	75.29 ± 0.20
3.0	96	3.5	0.75	200	41226 ± 1000	-	150.37 ± 1.87	75.18 ± 0.93	148.39 ± 3.61	74.20 ± 1.80
2.0	144	1.7	0.75	200	57921 ± 2561	436.46 ± 14.13	139.39 ± 1.94	139.39 ± 1.94	104.25 ± 4.61	104.25 ± 4.61
2.0	144	3.5	0.75	200	62305 ± 422.0	1072.6 ± 38.65	282.73 ± 4.02	141.37 ± 2.01	224.28 ± 1.52	112.14 ± 0.76
2.0	144	5.0	0.75	200	64005 ± 339.0	1668.9 ± 68.39	442.26 ± 2.82	147.42 ± 0.94	345.59 ± 1.82	115.20 ± 0.61
2.0	144	6.5	0.75	200	64527 ± 406.0	2740.1 ± 96.95	630.19 ± 0.93	157.55 ± 0.23	464.59 ± 2.95	116.14 ± 0.73
2.0	144	3.5	0.25	200	60599 ± 900.0	3889.9 ± 111.4	259.53 ± 3.04	129.77 ± 1.52	218.13 ± 3.25	109.07 ± 1.62
2.0	144	3.5	0.50	200	62288 ± 1722	1917.0 ± 61.94	281.11 ± 3.85	140.56 ± 1.92	224.23 ± 6.19	112.11 ± 3.10
2.0	144	3.5	0.75	200	62305 ± 422.0	1072.6 ± 38.65	282.73 ± 4.02	141.37 ± 2.01	224.28 ± 1.52	112.14 ± 0.76
2.0	144	3.5	1.00	200	65250 ± 4250	853.73 ± 25.30	316.21 ± 2.00	158.11 ± 1.00	234.89 ± 15.3	117.44 ± 7.65
2.0	144	3.5	0.75	50	47237 ± 478.0	4450.0 ± 186.4	196.80 ± 1.87	98.40 ± 0.94	170.05 ± 1.72	85.02 ± 0.86
2.0	144	3.5	0.75	100	55049 ± 72.0	1971.2 ± 64.73	236.61 ± 6.33	118.30 ± 316	198.16 ± 0.26	99.08 ± 0.13
2.0	144	3.5	0.75	200	62305 ± 422.0	1072.6 ± 38.65	282.73 ± 4.02	141.37 ± 2.01	224.28 ± 1.52	112.14 ± 0.76
2.0	144	3.5	0.75	400	72156 ± 761.0	611.52 ± 31.25	323.26 ± 7.09	161.63 ± 3.55	259.74 ± 2.74	129.87 ± 1.37

*t_T_*: total operation time; *H*: bed height; *Q*: flow rate of the influent solution; *C_0_*: influent Cr(VI) concentration; *N_b_*: volumetric biosorption capacity; *t_50_*: experimental time needed to retain 50% of influent total chromium; *m_r_*: mass of total chromium or Cr(VI) removed by the fixed-bed column; *q_b_*: total chromium or Cr(VI) removal capacity.

A plausible explanatory basis for the increase in Cr(VI) removal capacity at lower influent pH values may be attributed to the strong electrostatic attraction between the positively charged ligands present on the QCS surface at lower influent pH values and the negatively charged Cr(VI) oxyanions. Furthermore, the reductive biotransformation of Cr(VI) to Cr(III) consumes protons, and consequently Cr(VI) removal is favored at lower pH values [2, 7, 12–15]. Conversely, with increasing influent pH values, the extent of Cr(VI) removal decreases, presumably because of an increase in competition between Cr(VI) oxyanions and OH^-^ ions for the same functional groups on QCS surface and an increase of the negative surface charge resulting in higher electrostatic repulsion between the QCS surface and Cr(VI) oxyanions [13].

Fig 2B reveals that the slope of the breakthrough curves for removal of total chromium is strongly dependent on influent Cr(VI) solution pH. In contrast to the findings for the removal of Cr(VI), the presence of total chromium was observed in the outlet effluent from the beginning of operation of the fixed-bed column system at all influent pH levels for which assays were performed. These differences highlight the significance of monitoring both total chromium and Cr(VI) in Cr(VI) removal treatment systems.

At all column operation times assayed, the effluent total chromium concentration was higher than that of effluent Cr(VI) concentration for influent solution pH values ranging from 1.0 to 2.5; additionally, the difference between these two concentrations decreased as the influent pH increased. Contrastingly, the difference between concentrations of effluent total chromium and effluent Cr(VI) was minimal at the influent solution pH of 3.0.

The differences found between effluent total chromium concentration and effluent Cr(VI) concentration are attributed to the existence of Cr(III) in the effluent aqueous phase, which was not detected in the influent Cr(VI) solution but was formed as a product of Cr(VI) bioreduction by reducing organic compounds contained in QCS [12]. The highest Cr(III) concentrations were obtained at the influent Cr(VI) solution pH of 1.0, and they decreased as the influent solution pH increased, which confirms that bioreduction of Cr(VI) to Cr(III) consumes protons [15]. It is known that acorn shells are composed of, among other chemical compounds, polyphenols, polysaccharides, and lignocellulose [31], which have been reported to be capable of reducing Cr(VI) to Cr(III) under acidic conditions [32] and which may be the QCS components that cause the bioreduction of the hexavalent to the trivalent chromium described herein.

Taken together, the above findings indicate clearly that QCS biomass is able to remove Cr(VI) present in liquid phase by means of a complex mixed mechanism that includes the biotransformation of Cr(VI) to Cr(III) and the biosorption of chromium ions and that the extent and rate of total chromium biosorption and Cr(VI) bioreduction are highly dependent on the influent pH. Likewise, it appears from the kinetic results that the chromium biosorbed to the QCS surface is in the trivalent oxidation state, which was subsequently verified by XPS analysis.

As shown in Table 1, the highest biosorption capacity of total chromium (110.35 mg g^−1^) was obtained at the influent pH of 2.0. In addition, as influent pH decreased from 2.0 to 1.0 and increased from 2.0 to 3.0, biosorption capacity of total chromium decreased from 110.35 to 86.75 mg g^−1^ and from 110.35 to 74.20 mg g^−1^, respectively. Furthermore, it was found that the difference between the removal capacities for Cr(VI) and total chromium of fixed QCS bed decreased from 94.81 to 0.98 mg g^−1^ as the influent pH increased from 1.0 to 3.0, which suggests that as the influent Cr(VI) solution pH decreased, the quantity of Cr(III) that was produced from the bioreduction of Cr(VI) and that was released to the liquid phase increased.

Contrastingly, the highest total chromium removal capacity of QCS was previously obtained at a Cr(VI) solution pH value of 4.0 when a pH-controlled batch system was used [12]. These differences in the optimal Cr(VI) solution pH for total chromium removal can be ascribed to the very different operating conditions for continuous and batch process systems. These results highlight the significance of ascertaining the most adequate solution pH for the removal of total chromium and Cr(VI) using the operation method to be employed on large scale processes [2]. Contrarily, most studies on total chromium removal performed in fixed-bed systems have used the Cr(VI) solution pH that was found as the optimum in batch systems [19, 33–35].

Based on the above results, further fixed-bed column studies were conducted at an influent Cr(VI) solution pH of 2.0. An optimum influent solution pH of 1.5 has been reported for removal of total chromium by a fixed bed of Hass avocado shell biosorbent [2].

### Effect of bed height (or bed depth) on removal of total chromium and Cr(VI)

The performance of QCS biomass in removing total chromium and Cr(VI) was assayed for various bed heights at 0.75 mL min^−1^ flow rate (398.23 L m^−2^ h^−1^) and 200 mg L^−1^ influent Cr(VI) concentration. In order to yield different bed heights, 1, 2, 3, and 4 g of QCS biomass were added to produce bed heights of 1.7, 3.5, 5.0, and 6.5 cm, respectively.

**Fig 3:**
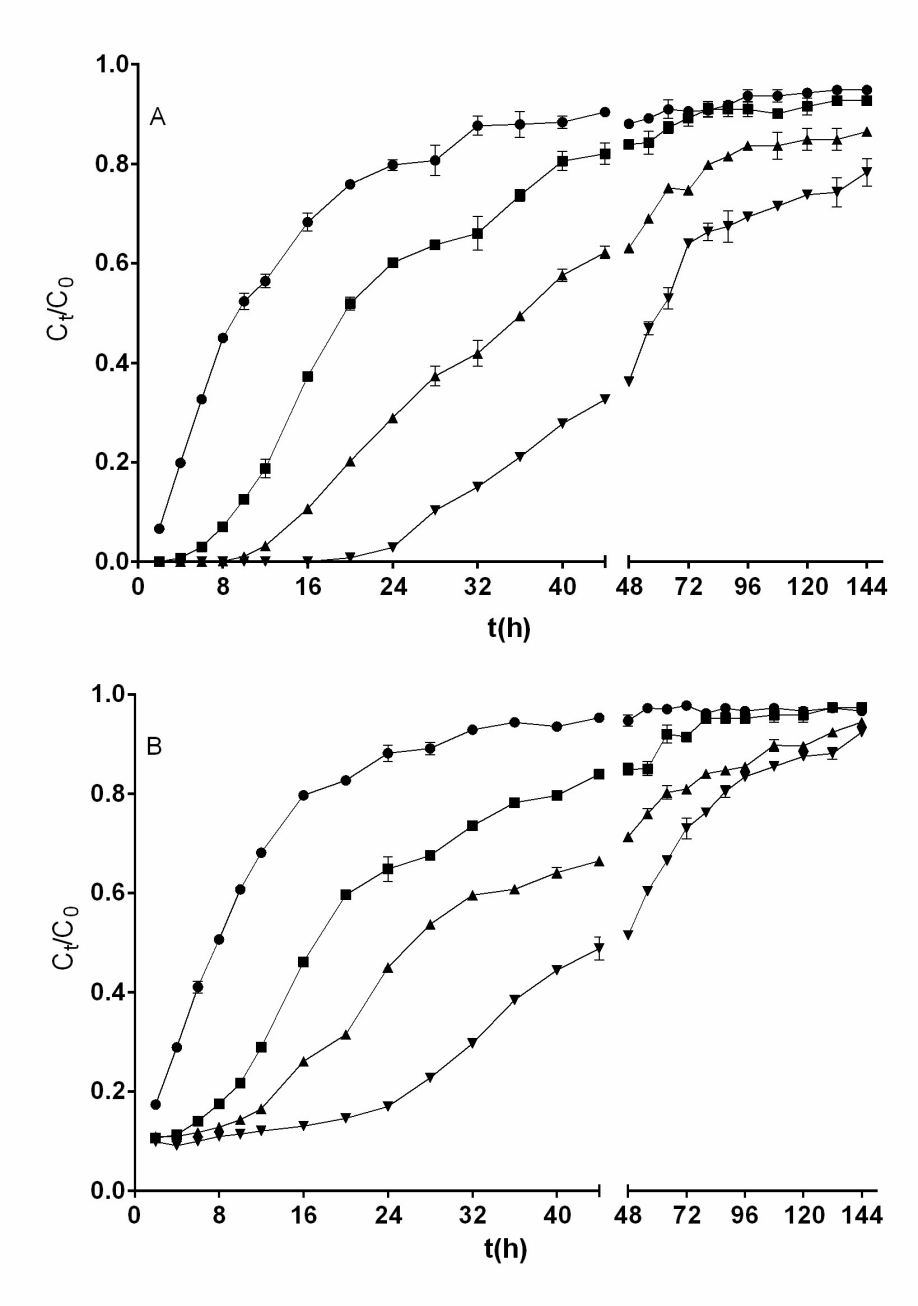
**Influence of bed height on the breakthrough curve for removal of Cr(VI) (A) and total chromium (B) by QCS [bed height (cm): ●, 1.7; ■, 3.5; ▲, 5; ▼, 6.5].** Conditions: influent Cr(VI) concentration, 200 mg L^−1^; influent flow rate, 0.75 mL min^−1^; solution pH, 2.0. When they do not appear, standard deviation bars are smaller than the size of the symbols.

The breakthrough profiles of the removal of total chromium and Cr(VI) at the various bed heights are shown in Fig 3. It is apparent that the breakthrough curves become steeper as the bed height decreases, which indicates that the total chromium and Cr(VI) concentrations in the effluent increased, that bed exhaustion was reached faster, and that less total chromium and Cr(VI) (*m*_r_) were removed as the bed height decreased (Table 1).

When a bed height of 1.7 cm (1 g) was used, Cr(VI) was found in the outlet effluent from the beginning of the continuous operation. Contrastingly, Cr(VI) was not present in the outlet effluent in the first 4, 8, and 16 h of operation of the fixed-bed column with bed heights of 3.5, 5.0, and 6.5 cm (2, 3, and 4 g), respectively.

Contrary to what was observed in the removal of Cr(VI), total chromium was present in the outlet effluent from the beginning of continuous fixed-bed column operation at all bed heights assayed. Furthermore, effluent total chromium concentration was higher than effluent Cr(VI) concentration at all biosorbent bed heights and operation times assayed, which is ascribed to the bioreduction of Cr(VI) to Cr(III). The differences found in effluent Cr(VI) and total chromium concentrations highlight the significance of measuring both total chromium and Cr(VI) in chromium removal treatment processes.

Similar qualitative kinetics have previously been reported for Cr(VI) removal in a continuous fixed-bed column with immobilized *Spirulina platensis* [34] as well as for total chromium removal by a bed packed with a modified and immobilized *Auricularia auricula* spent substrate [36].

Table 1 shows that the mass of total chromium and Cr(VI) removed (*m*_r_) by the QCS bed increased as the bed height increased, and this may be due to the increase in the amount of biosorbent used in the bed, which increases the surface area through the bed and the number of active sites for total chromium biosorption and Cr(VI) bioreduction [37]. Furthermore, the increase in bed height resulted in a longer residence time of the metal solution in the column, allowing the chromium ions to be in contact with the biosorbent for a longer period as well as for the chromium ions to diffuse more deeply inside the QCS biosorbent, which led to the removal of a larger mass of total chromium and Cr(VI) by the QCS bed [17, 36]. However, QCS's capacity for removing total chromium and Cr(VI) increased only slightly with the increase in bed height (Table 1). These results indicate that the amount of total chromium and Cr(VI) removed was almost directly proportional to the amount of QCS biosorbent available in the bed. A similar relationship between total chromium removal capacity and bed height was reported by Senthilkumar et al. [35] and Zang et al. [36], who used beds packed with *Sargassum polycystum* and with *Auricularia auricula* chemically modified with cetyltrimethyl ammonium bromide and immobilized in alginate beads, respectively.

### Influence of flow rate on removal of total chromium and Cr(VI)

Flow rate is an important parameter affecting the performance of a continuous biosorption process for the removal of heavy metals from aqueous solutions as it determines the length of time the heavy metal is in contact with the solution [28]. Therefore, the effect of flow rate on the removal of total chromium and Cr(VI) by QCS was assessed by modifying the flow rate from 0.25 to 1 mL min^−1^ (corresponding to volumetric fluxes of 132.74 to 530.97 L m^−2^ h^−1^) and keeping constant the influent Cr(VI) concentration (200 mg L^−1^), influent solution pH (2.0), and bed height (3.5 cm).

**Fig 4:**
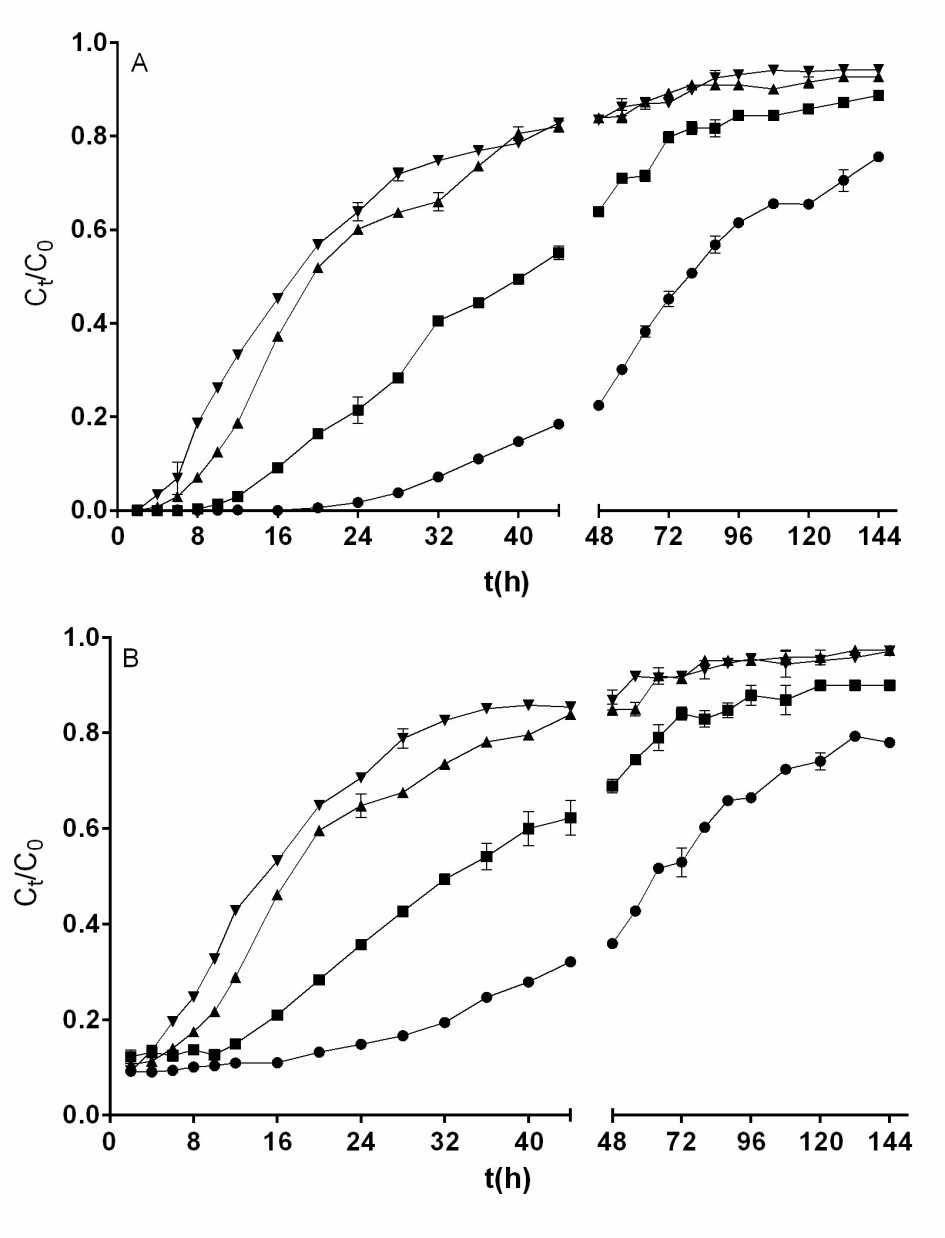
**Influence of influent flow rate on the breakthrough curve for removal of Cr(VI) (A) and total chromium (B) by QCS [flow rate (mL min**^**-1**^**): ●, 0.25; ■, 0.5; ▲, 0.75; ▼, 1.0].** Conditions: influent Cr(VI) concentration, 200 mg L^−1^; bed height, 3.5 cm; solution pH, 2.0. When they do not appear, standard deviation bars are smaller than the size of the symbols.

The effect of flow rate on breakthrough performance for the removal of total chromium and Cr(VI) is shown in Fig 4. It can be seen that no Cr(VI) was found in the outlet effluent in the first 2, 4, 8, and 16 h of operation of the fixed-bed column with inlet solution flow rates of 1.0, 0.75, 0.5, and 0.25 mL min^−1^, respectively (Fig 4A), which indicates that the service time of the fixed bed for Cr(VI) removal increased as the flow rate decreased. At later times of operation of the fixed-bed column, the Cr(VI) concentration in the outlet effluent increased with the increasing of the flow rate, indicating a diminution in the capacity for Cr(VI) removal by QCS bed due to the gradual depletion of active sites for chromium biosorption and Cr(VI) bioreduction. Similar kinetic profiles for Cr(VI) removal have been reported in the literature [21, 22, 34, 38].

At all the inlet flow rates assayed, total chromium was found in the outlet effluent from the beginning of fixed-bed column operation (Fig 4B), even at operation times when no Cr(VI) was present in the effluent (Fig 4A), indicating that the chromium present in the effluent was in the trivalent form.

Furthermore, as the flow rate increased, total chromium concentration in the effluent increased rapidly, resulting in much sharper breakthrough curves. This behavior is due to the fact that at higher inlet flow rates the duration of contact between chromium ions and the biosorbent surface is shorter, and hence the chromium ions have less time to bind to the active biosorption sites on the biosorbent surface or diffuse into the pores of the biosorbent, leaving the fixed-bed column at higher concentrations and before the biosorption equilibrium occurs [16, 21].

Qualitatively similar breakthrough curves were reported for total chromium removal by polysulfone-immobilized *Aeromonas hydrophila* [19] and by *Auricularia auricula* modified with cetyltrimethyl ammonium bromide and immobilized in alginate beads [36], and for Cr(III) removal by olive stone [28].

Only a small increase in Cr(VI) removal capacity (from 129.77 to 158.11 mg g^−1^) and total chromium removal capacity (from 109.07 to 117.44 mg g^−1^) was found with the increase in flow rate (from 0.25 to 1.0 mL min^−1^). This is attributed to the fact that the influent flow rate has two opposing effects on the removal of a target contaminant: the higher the flow rate, the shorter the duration of contact between the chromium ions and the biosorbent (which decreases the removal capacity), but the higher the rate of mass transfer of chromium ions between liquid and solid phases (which increases the removal capacity) in a fixed-bed column [30].

An increase in removal capacity of Cr(VI) with an increase in inlet flow rate was reported by Gokhale et al. [34] in their fixed-bed column studies using calcium alginate-immobilized *Spirulina platensis* as biosorbent. Likewise, Vieira et al. [30] found that the total chromium biosorption capacity of a column packed with *Sargassum* sp. biosorbent increased as the flow rate increased. In contrast, the opposite effect for inlet flow rate on total chromium biosorption capacity was observed in fixed-bed column studies using *Sargassum polycystum* [35], and *Auricularia auricula* modified by cetyltrimethyl ammonium bromide and immobilized by alginate [36].

It is worth noting that the difference between removal capacities of Cr(VI) and total chromium of fixed-bed QCS increased from 20.7 to 40.67 mg g^−1^ as the influent flow rate increased from 0.25 to 1.0 mL min^−1^, which suggests that as the influent flow rate increased, the quantity of Cr(III) that was produced from the bioreduction of Cr(VI) and that was released to the liquid phase increased.

### Influence of influent Cr(VI) concentration on removal of total chromium and Cr(VI)

**Fig 5:**
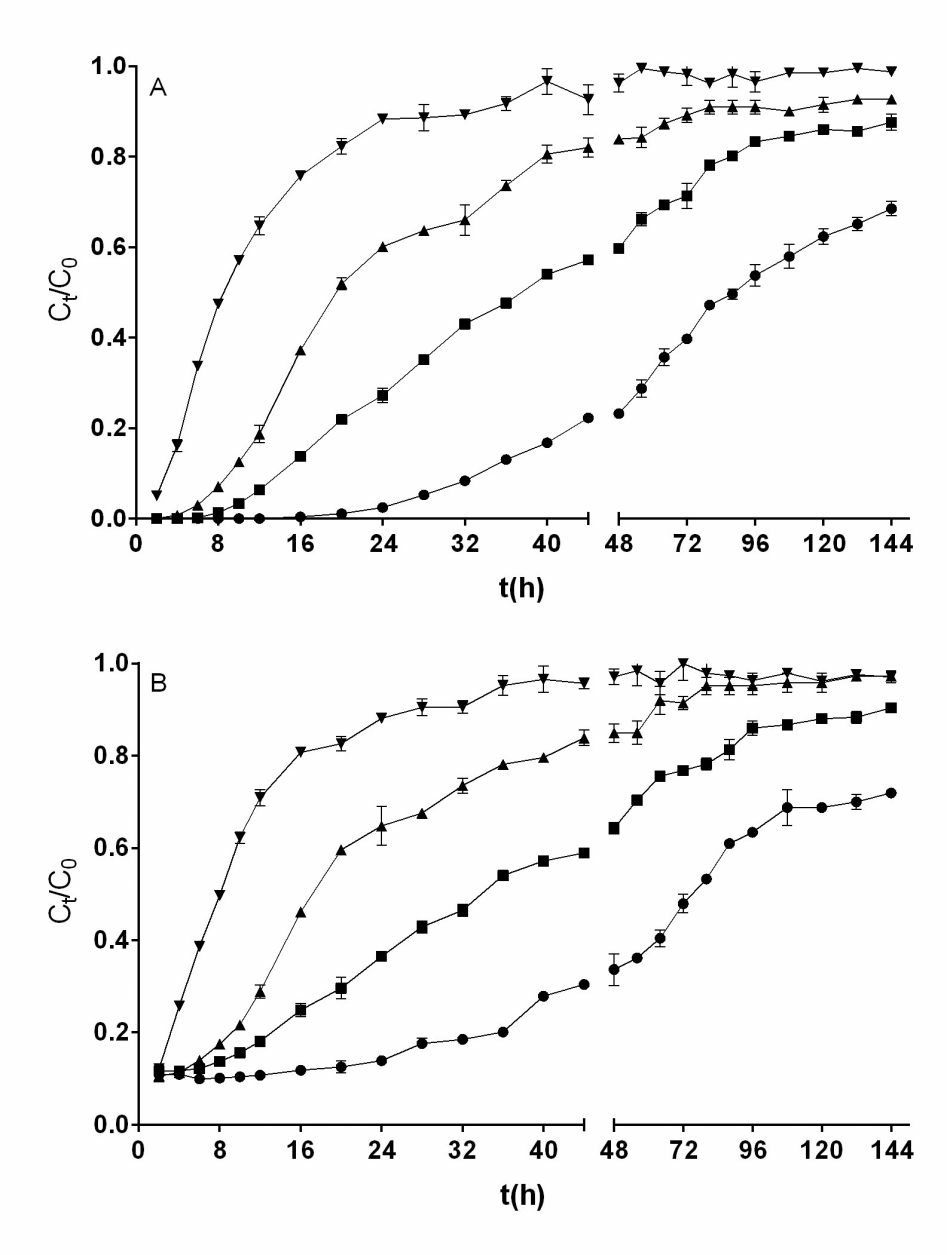
**Influence of influent Cr(VI) concentration on the breakthrough curve for removal of Cr(VI) (A) and total chromium (B) by QCS [Cr(VI) concentration (mg L**^**-1**^**): ●, 50; ■, 100; ▲, 200; ▼, 400].** Conditions: influent flow rate, 0.75 mL min^−1^; bed height, 3.5 cm; solution pH, 2.0. When they do not appear, standard deviation bars are smaller than the size of the symbols.

Influent metal ion concentration is among the important parameters affecting the removal performance of the biosorbent packed in the column because it provides the driving force of the chromium concentration gradient for the mass transfer of chromium ions from the aqueous solution to the QCS surface [17], and hence it should be investigated in all fixed-bed biosorption studies. The effect of influent Cr(VI) concentration (50, 100, 200, and 400 mg L^−1^) on breakthrough curves at a constant bed height of 3.5 cm, influent flow rate of 0.75 mL min^−1^, and influent solution pH of 2.0 is shown in Fig 5. Results show that the shape and gradient of the breakthrough curves for removal of Cr(VI) (Fig 5A) and total chromium (Fig 5B) differed considerably with the variation in the influent Cr(VI) concentration.

Similar to what was observed in the studies of the effect of influent solution pH, biosorbent bed height, and inlet flow rate in the fixed-bed column, the lowest concentrations of total chromium and Cr(VI) in the effluent were quantified in the first hours of continuous column operation. Thereafter, total chromium and Cr(VI) concentrations increased gradually with operation time, suggesting a decrease in capacity for removal of total chromium and Cr(VI) due to the progressive exhaustion of active sites for chromium biosorption and Cr(VI) bioreduction [30].

It is apparent from Fig 5A that when the influent Cr(VI) concentration was 50, 100, or 200 mg L^−1^, all the Cr(VI) present in the influent solution was removed in the first hours of column operation. In contrast, at all influent Cr(VI) concentrations assayed, total chromium was found in the outlet effluent from the beginning of the continuous operation. The fact that during the first hours of column operation with influent Cr(VI) concentrations ranging from 50 to 200 mg L^−1^, total chromium but not Cr(VI) was present in the effluent indicates the presence of Cr(III) in the effluent.

As the influent Cr(VI) concentration increases, the breakthrough curves exhibit a steeper slope, meaning that the effluent total chromium and Cr(VI) concentrations increased, column saturation was reached more rapidly, and the column operation time decreased. These results are in agreement with those reported for Cr(VI) removal [21, 34, 38] and total chromium biosorption [30, 33] in continuous fixed-bed systems.

With the increase in influent Cr(VI) concentration from 50 to 400 mg L^−1^, the capacities for removal of Cr(VI) and total chromium increased from 98.40 to 161.63 mg g^−1^ and from 85 to 129.87 mg g^−1^, respectively (Table 1). These increases may be explained by the fact that the higher the influent Cr(VI) concentration, the higher the number of chromium ions in the aqueous phase and consequently the higher the enhancement of interactions between the chromium ions and the QCS biosorbent. A higher influent Cr(VI) concentration also increases the chromium concentration gradient, which causes a faster transport of chromium ions from the bulk of the aqueous solution to the biosorbent surface due to an increased diffusion coefficient or mass transfer coefficient [7, 38], as well as strengthening the thermodynamic driving force that overcomes all the mass transfer resistance of chromium ions from the aqueous solution to the QCS biomass [7, 21]. An increase in removal capacity of total chromium with an increase in influent Cr(VI) concentration was also informed by Vieira et al. [30] and Zang et al. [36], who used columns packed with *Sargassum* sp. and with *Auricularia auricula* chemically modified with cetyltrimethyl ammonium bromide and immobilized in alginate beads, respectively.

Additionally, it was found that the difference between the capacities for removal of Cr(VI) and total chromium by fixed QCS bed increased from 13.38 to 31.76 mg g^−1^ as the influent Cr(VI) concentration increased from 50 to 400 mg L^−1^, indicating that the quantity of Cr(III) that was produced from the bioreduction of Cr(VI) and that was released to the liquid phase increased as the influent Cr(VI) concentration increased.

For the first time the above results clearly show that the kinetic profiles of total chromium and Cr(VI) removal from Cr(VI) aqueous solutions at different operational conditions (influent pH levels, influent flow rates, influent Cr(VI) concentrations, and biosorbent bed heights) in a fixed-bed column vary, and that these differences are due to the reductive biotransformation of Cr(VI) to Cr(III).

### Modeling of total chromium biosorption breakthrough curves

Modeling of breakthrough curves plays a key role in analyzing and explaining experimental dynamic biosorption column data, identifying mechanisms relevant to the biosorption process, and predicting changes due to different operating conditions as well as in the design, optimization, and scale-up of biosorption processes [2, 33].

In this work, Thomas, Bohart–Adams, Yan, dose-response, and Yoon–Nelson models were developed to identify the best model(s) for predicting the dynamic behavior of the fixed-bed column with respect to influent Cr(VI) solution pH, influent flow rate, bed height, and influent Cr(VI) concentration. Tables 2–6 show the kinetic parameter values of the breakthrough curve models, along with the corresponding values of the error functions (*r*^2^, RMSE, SSE, AIC, and 95% confidence interval) applied in this work.

**Table 2:** Bohart–Adams model parameters for total chromium biosorption onto QCS under different operational conditions.

pH	*t_T_ (h)*	*H (cm)*	*Q (mL min^-1^)*	*C_0_ (mg L^-1^)*	*k_AB_* (x 10^-6^) (L mg^-1^ min^-1^)	*N_0_* (mg L^-1^)	*r^2^*	*SSE*	*RMSE*	*AIC*
1.0	96	3.5	0.75	200	5.878 ± 0.524	45512 ± 2335	0.9600	0.1491	0.04984	-367.5
1.5	96	3.5	0.75	200	8.971 ± 1.008	40424 ± 2328	0.9497	0.2483	0.06380	-342.4
2.0	96	3.5	0.75	200	7.420 ± 0.803	53237 ± 2814	0.9541	0.2685	0.06689	-331.0
2.5	96	3.5	0.75	200	10.05 ± 0.756	38241 ± 1435	0.9801	0.1087	0.04222	-394.4
3.0	96	3.5	0.75	200	2.335 ± 0.155	44983 ± 2056	0.9666	0.0310	0.02293	-456.2
2.0	144	1.7	0.75	200	13.52 ± 1.380	46326 ± 2323	0.9650	0.1407	0.04390	-464.6
2.0	144	3.5	0.75	200	7.436 ± 0.760	53196 ± 2653	0.9600	0.2639	0.06323	-371.1
2.0	144	5.0	0.75	200	4.624 ± 0.504	56841 ± 3039	0.9410	0.3963	0.07368	-386.9
2.0	144	6.5	0.75	200	3.731 ± 0.252	64229 ± 2026	0.9746	0.1788	0.04949	-446.6
2.0	144	3.5	0.25	200	2.470 ± 0.153	60332 ± 1819	0.9670	0.1582	0.04687	-448.6
2.0	144	3.5	0.50	200	4.339 ± 0.383	64433 ± 2734	0.9589	0.2443	0.06039	-383.0
2.0	144	3.5	0.75	200	7.436 ± 0.760	53196 ± 2653	0.9600	0.2639	0.06323	-371.1
2.0	144	3.5	1.00	200	8.673 ± 0.895	56661 ± 2947	0.9581	0.2502	0.06066	-388.0
2.0	144	3.5	0.75	50	9.706 ± 0.464	46664 ± 1075	0.9559	0.1508	0.04780	-466.0
2.0	144	3.5	0.75	100	7.220 ± 0.636	48947 ± 2251	0.9535	0.2802	0.06196	-412.9
2.0	144	3.5	0.75	200	7.436 ± 0.760	53196 ± 2653	0.9600	0.2639	0.06323	-371.1
2.0	144	3.5	0.75	400	4.405 ± 0.458	67139 ± 3781	0.9574	0.2189	0.05475	-431.4

*t_T_*: total operation time; *H*: bed height; *Q*: influent solution flow rate; *C_0_*: influent Cr(VI) concentration; *k_AB_*: kinetic constant of the Bohart-Adams model; *N_0_*: volumetric biosorption capacity.

**Table 3:** Thomas model parameters for total chromium biosorption onto QCS under different operational conditions.

pH	*t_T_ (h)*	*H (cm)*	*Q (mL min^-1^)*	*C_0_ (mg L^-1^)*	k_Th_ (x 10^-3^) (mL min^-1^ mg^-1^)	q_Th_ (mg g^-1^)	*r^2^*	*SSE*	*RMSE*	*AIC*
1.0	96	3.5	0.75	200	5.707 ± 0.508	71.47 ± 4.875	0.9600	0.1491	0.04984	-367.5
1.5	96	3.5	0.75	200	8.971 ± 1.008	70.51 ± 4.530	0.9497	0.2483	0.06380	-342.4
2.0	96	3.5	0.75	200	7.420 ± 0.803	98.20 ± 5.605	0.9541	0.2685	0.06689	-331.0
2.5	96	3.5	0.75	200	10.05 ± 0.756	68.52 ± 2.810	0.9801	0.1087	0.04222	-394.4
3.0	96	3.5	0.75	200	2.335 ± 0.155	47.03 ± 10.42	0.9666	0.0310	0.02293	-456.2
2.0	144	1.7	0.75	200	13.52 ± 1.380	76.98 ± 4.620	0.9650	0.1407	0.04390	-464.6
2.0	144	3.5	0.75	200	7.436 ± 0.760	98.16 ± 5.320	0.9600	0.2639	0.06323	-371.1
2.0	144	5.0	0.75	200	4.624 ± 0.504	98.40 ± 5.595	0.9410	0.3963	0.07368	-386.9
2.0	144	6.5	0.75	200	3.731 ± 0.252	113.3 ± 3.750	0.9746	0.1788	0.04949	-446.6
2.0	144	3.5	0.25	200	2.470 ± 0.153	109.9 ± 3.300	0.9670	0.1582	0.04687	-448.6
2.0	144	3.5	0.50	200	4.434 ± 0.358	103.3 ± 5.000	0.9589	0.2443	0.06039	-383.0
2.0	144	3.5	0.75	200	7.420 ± 0.803	98.16 ± 5.320	0.9600	0.2639	0.06323	-371.1
2.0	144	3.5	1.00	200	8.675 ± 0.914	103.8 ± 5.950	0.9581	0.2502	0.06066	-388.0
2.0	144	3.5	0.75	50	9.706 ± 0.464	86.57 ± 1.910	0.9559	0.1508	0.04780	-466.0
2.0	144	3.5	0.75	100	7.220 ± 0.636	88.32 ± 4.260	0.9535	0.2802	0.06196	-412.9
2.0	144	3.5	0.75	200	7.436 ± 0.760	98.14 ± 5.275	0.9600	0.2639	0.06323	-371.1
2.0	144	3.5	0.75	400	4.405 ± 0.458	113.3 ± 7.750	0.9574	0.2189	0.05475	-431.4

*t_T_*: total operation time; *H*: bed height; *Q*: influent solution flow rate; *C_0_*: influent Cr(VI) concentration; *k_Th_*: Thomas model rate constant; *q_Th_*: concentration of total chromium in the biosorbent.

**Table 4:** Yoon–Nelson model parameters for total chromium biosorption onto QCS under different operational conditions.

pH	*t_T_ (h)*	*H (cm)*	*Q (mL min^-1^)*	*C_0_ (mg L^-1^)*	*k_YN_ (x 10^-3^) (L mg^-1^ min^-1^)*	*τ (min)*	*r^2^*	*SSE*	*RMSE*	*AIC*
1.0	96	3.5	0.75	200	1.178 ± 0.105	922.9 ± 62.96	0.9600	0.1491	0.04984	-367.5
1.5	96	3.5	0.75	200	1.833 ± 0.206	927.8 ± 59.64	0.9497	0.2483	0.06380	-342.4
2.0	96	3.5	0.75	200	1.530 ± 0.157	1260 ± 70.50	0.9541	0.2685	0.06689	-331.0
2.5	96	3.5	0.75	200	2.001 ± 0.151	915.8 ± 37.52	0.9801	0.1087	0.04222	-394.4
3.0	96	3.5	0.75	200	0.471 ± 0.031	624.6 ± 138.2	0.9666	0.0310	0.02293	-456.2
2.0	144	1.7	0.75	200	2.719 ± 0.278	511.7 ± 32.75	0.9650	0.1407	0.04390	-464.6
2.0	144	3.5	0.75	200	1.533 ± 0.157	1259 ± 68.00	0.9600	0.2639	0.06323	-371.1
2.0	144	5.0	0.75	200	0.927 ± 0.101	1975 ± 112.2	0.9410	0.3963	0.07368	-386.9
2.0	144	6.5	0.75	200	0.739 ± 0.050	3061 ± 97.50	0.9746	0.1788	0.04949	-446.6
2.0	144	3.5	0.25	200	0.503 ± 0.031	4368 ± 132.0	0.9670	0.1582	0.04687	-448.6
2.0	144	3.5	0.50	200	0.897 ± 0.079	2193 ± 96.00	0.9589	0.2443	0.06039	-383.0
2.0	144	3.5	0.75	200	1.533 ± 0.157	1259 ± 68.00	0.9600	0.2639	0.06323	-371.1
2.0	144	3.5	1.00	200	1.775 ± 0.183	1015 ± 58.45	0.9581	0.2502	0.06066	-388.0
2.0	144	3.5	0.75	50	0.486 ± 0.023	4677 ± 103.5	0.9559	0.1508	0.04780	-466.0
2.0	144	3.5	0.75	100	0.721 ± 0.063	2368 ± 114.0	0.9535	0.2802	0.06196	-412.9
2.0	144	3.5	0.75	200	1.533 ± 0.157	1259 ± 68.00	0.9600	0.2639	0.06323	-371.1
2.0	144	3.5	0.75	400	1.766 ± 0.184	755.8 ± 51.70	0.9574	0.2189	0.05475	-431.4

*t_T_*: total operation time; *H*: bed height; *Q*: influent solution flow rate; *C_0_*: influent Cr(VI) concentration; *k_YN_*: Yoon-Nelson model rate constant; *τ*: operation time needed to reach a ratio of effluent to influent total chromium concentration equal to 0.5.

**Table 5:** Dose–response model parameters for total chromium biosorption onto QCS under different operational conditions.

pH	*t_T_ (h)*	*H (cm)*	*Q (mL min^-1^)*	*C_0_ (mg L^-1^)*	*α (dimensionless)*	*β (min)*	*r^2^*	*SSE*	*RMSE*	*AIC*
1.0	96	3.5	0.75	200	1.213± 0.110	716.4 ± 56.75	0.9429	0.2128	0.05955	-345.4
1.5	96	3.5	0.75	200	1.624 ± 0.106	786.1 ± 36.40	0.9772	0.1123	0.04291	-392.3
2.0	96	3.5	0.75	200	1.766 ± 0.090	1075 ± 35.00	0.9882	0.0690	0.03392	-415.2
2.5	96	3.5	0.75	200	1.821 ± 0.117	785.7 ± 32.35	0.9815	0.1014	0.04077	-398.8
3.0	96	3.5	0.75	200	0.528 ± 0.072	140.2 ± 40.15	0.8126	0.1741	0.05433	-351.0
2.0	144	1.7	0.75	200	1.545 ± 0.059	435.7 ± 12.55	0.9915	0.0343	0.02166	-570.5
2.0	144	3.5	0.75	200	1.781 ± 0.086	1074 ± 33.50	0.9894	0.0700	0.03258	-461.3
2.0	144	5.0	0.75	200	1.592 ± 0.074	1675 ± 50.50	0.9876	0.0834	0.03381	-503.7
2.0	144	6.5	0.75	200	2.005 ± 0.128	2739 ± 86.50	0.9794	0.1446	0.04451	-462.5
2.0	144	3.5	0.25	200	1.698 ± 0.110	3888 ± 131.0	0.9723	0.1330	0.04297	-461.5
2.0	144	3.5	0.50	200	1.693 ± 0.117	1908 ± 76.00	0.9746	0.1507	0.04743	-416.4
2.0	144	3.5	0.75	200	1.781 ± 0.086	1074 ± 33.50	0.9894	0.0700	0.03258	-461.3
2.0	144	3.5	1.00	200	1.675 ± 0.065	854.3 ± 22.95	0.9924	0.0456	0.02589	-507.2
2.0	144	3.5	0.75	50	1.532 ± 0.136	4424 ± 212.5	0.9604	0.1418	0.04533	-435.0
2.0	144	3.5	0.75	100	1.471 ± 0.065	1961 ± 57.50	0.9874	0.0761	0.03227	-510.6
2.0	144	3.5	0.75	200	1.781 ± 0.086	1074 ± 33.50	0.9894	0.0700	0.03258	-461.3
2.0	144	3.5	0.75	400	1.489 ± 0.059	603.9 ± 18.60	0.9910	0.0465	0.02525	-547.5

*t_T_*: total operation time; *H*: bed height; *Q*: influent solution flow rate; *C_0_*: influent Cr(VI) concentration; α and β: dose-response model constants.

**Table 6:** Yan model parameters for total chromium biosorption onto QCS under different operational conditions.

pH	*t_T_ (h)*	*H (cm)*	*Q (mL min^-1^)*	*C_0_ (mg L^-1^)*	*α (dimensionless)*	*q_0_ (mg g^-1^)*	*r^2^*	*SSE*	*RMSE*	*AIC*
1.0	96	3.5	0.75	200	1.213± 0.110	55.48 ± 4.390	0.9429	0.2128	0.05955	-345.4
1.5	96	3.5	0.75	200	1.624 ± 0.106	59.75 ± 2.765	0.9772	0.1123	0.04291	-392.3
2.0	96	3.5	0.75	200	1.766 ± 0.090	83.79 ± 2.720	0.9882	0.0690	0.03392	-415.2
2.5	96	3.5	0.75	200	1.821 ± 0.117	58.79 ± 2.425	0.9815	0.1014	0.04077	-398.8
3.0	96	3.5	0.75	200	0.528 ± 0.072	10.55 ± 3.024	0.8126	0.1741	0.05433	-351.0
2.0	144	1.7	0.75	200	1.545 ± 0.059	65.53 ± 1.895	0.9915	0.0343	0.02166	-570.5
2.0	144	3.5	0.75	200	1.781 ± 0.086	83.13 ± 2.600	0.9894	0.0700	0.03258	-461.3
2.0	144	5.0	0.75	200	1.592 ± 0.074	83.49 ± 2.530	0.9876	0.0834	0.03381	-503.7
2.0	144	6.5	0.75	200	2.005 ± 0.128	101.4 ± 3.195	0.9794	0.1446	0.04451	-462.5
2.0	144	3.5	0.25	200	1.698 ± 0.110	97.81 ± 3.295	0.9723	0.1330	0.04297	-461.5
2.0	144	3.5	0.50	200	1.693 ± 0.117	98.53 ± 3.950	0.9746	0.1507	0.04743	-416.4
2.0	144	3.5	0.75	200	1.781 ± 0.086	93.73 ± 2.600	0.9894	0.0700	0.03258	-461.3
2.0	144	3.5	1.00	200	1.675 ± 0.065	87.30 ± 2.345	0.9924	0.0456	0.02589	-507.2
2.0	144	3.5	0.75	50	1.532 ± 0.136	81.89 ± 3.930	0.9604	0.1418	0.04533	-435.0
2.0	144	3.5	0.75	100	1.471 ± 0.065	83.15 ± 2.135	0.9874	0.0761	0.03227	-510.6
2.0	144	3.5	0.75	200	1.781 ± 0.086	83.73 ± 2.600	0.9894	0.0700	0.03258	-461.3
2.0	144	3.5	0.75	400	1.489 ± 0.059	90.55 ± 2.795	0.9910	0.0465	0.02525	-547.5

*t_T_*: total operation time; *H*: bed height; *Q*: influent solution flow rate; *C_0_*: influent Cr(VI) concentration; α: Yan model constant; *q_0_*: maximum biosorption capacity.

Results show that the fitness of the breakthrough models to the data of total chromium biosorption depends on the pH of the influent Cr(VI) solution. The Yoon–Nelson, Thomas, and Bohart–Adams models provided higher values of *r*^2^, and lower values of SSE, RMSE, and AIC, than the dose–response and Yan models at influent pH values of 1.0 and 3.0. Contrastingly, at influent pH values of 1.5, 2.0, and 2.5, the dose–response and Yan models exhibited higher *r*^2^ values and lower SSE, RMSE, and AIC values than the Yoon–Nelson, Thomas, and Bohart–Adams models. The differences are attributed to the different symmetry and shape of the chromium breakthrough curves obtained at influent pH values of 1.0 and 3.0 compared with those obtained at pH 1.5, 2.0, and 2.5.

In spite of the fact that they rendered high *r*^2^ values and low SSE, RMSE, and AIC values at pH 1.0 and 3.0, the Thomas and Yoon–Nelson models were not capable of predicting the experimental data of total chromium biosorption capacity and the time needed to reach a ratio of effluent to influent total chromium concentration equal to 0.5, respectively, at all the influent solution pH values for which assays were performed. Therefore, the Thomas and Yoon–Nelson models are not adequate to describe the total chromium biosorption performance of QCS in the fixed-bed column at the influent solution pH values for which assays were performed. In contrast, the Bohart–Adams model predicted maximum volumetric biosorption capacities (*N*_0_; Table 2) that are in good agreement with the experimental values (*N*_b_; Table 1) obtained at influent pH values of 1.0 and 3.0 (p > 0.05). The significance of the Bohart–Adams model is its simplicity and its assumptions that intraparticle diffusion and external mass transfer resistance are negligible and that biosorption kinetics is controlled by the surface chemical reaction between the adsorbate and the biosorbent [28].

Likewise, although very high *r*^2^ values and low AIC, SSE, and RMSE values were obtained with the Yan model at influent pH values of 1.5, 2.0, and 2.5, it was not able to accurately predict the experimental total chromium biosorption capacities of QCS at all the influent pH values for which assays were performed (Tables 1 and 6). Consequently, the Yan model is not suitable for describing the kinetic behavior of total chromium biosorption onto QCS in the fixed-bed column at these influent solution pH values. In contrast, no statistically significant differences (p > 0.05) were found between the experimental values (*t*_50_; Table 1) and those predicted by the dose–response model (*β*; Table 5) for the time needed to retain 50% of inlet total chromium at pH values in the range from 1.0 to 2.5. Hence, the dose–response model exhibited a satisfactory ability to predict the breakthrough curves for total chromium removal obtained within the influent pH range of 1.0 to 2.5. The dose–response model is frequently employed to describe pharmacokinetic processes and is presently applied to describe the kinetic behavior of fixed-bed biosorption columns [28].

The above results show clearly that the fitness of the breakthrough curve models to the experimental kinetic biosorption data vary with influent solution pH (i.e., different kinetic models best describe the dynamic behavior of total chromium biosorption at different influent pH values). These results are in concordance with the findings of Aranda-García and Cristiani-Urbina [2], who examined the influence of solution pH on the removal of total chromium and Cr(VI) from aqueous solutions by Hass avocado shell using a continuous fixed-bed column system.

Likewise, the Yan and dose–response models provided the highest *r*^2^ and the lowest RMSE, SSE, and AIC values of the five assayed kinetic models at all QCS bed heights, influent flow rates, and influent Cr(VI) concentrations (Tables 2–6). However, the Yan model was not able to forecast the experimental data of total chromium biosorption capacity at the different biosorbent bed heights, influent flow rates, and influent Cr(VI) concentrations assayed (Tables 1 and 6), and consequently, the Yan model is not suitable for describing the dynamic behavior of total chromium biosorption onto QCS in the continuous fixed-bed reactor. Contrastingly, the dose–response model accurately predicted the time (*β*) needed to reach a *C_t_*/*C*_0_ ratio equal to 0.5 (*t*_50_ and *β* in Tables 1 and 5), and consequently this model shows good agreement with the experimental breakthrough curves obtained at the bed heights, feed flow rates, and inlet Cr(VI) concentrations tested. As expected, the *β* value predicted by the dose–response model increased as the bed height increased and as the influent flow rate and influent Cr(VI) concentration decreased.

Moreover, the dose–response model adequately represents the experimental kinetic data of Cr(III) biosorption onto olive stone [28, 37] and those of Cd(II) onto orange peels [27].

### Mechanism of Cr(VI) removal from aqueous solution by QCS

The results from the kinetic studies conducted in the present work showed clearly that both Cr(III) and Cr(VI) were present in the outlet effluent solution from the fixed-bed column. As Cr(III) was not present in the influent Cr(VI) solution, its presence in the effluent indicates that QCS is capable of biotransforming Cr(VI) to Cr(III). To establish the mechanism responsible for removal of Cr(VI) from aqueous solution by QCS, it is necessary to characterize the redox state of the chromium ions stick to the biosorbent, and XPS was employed here for this purpose.

**Fig 6:**
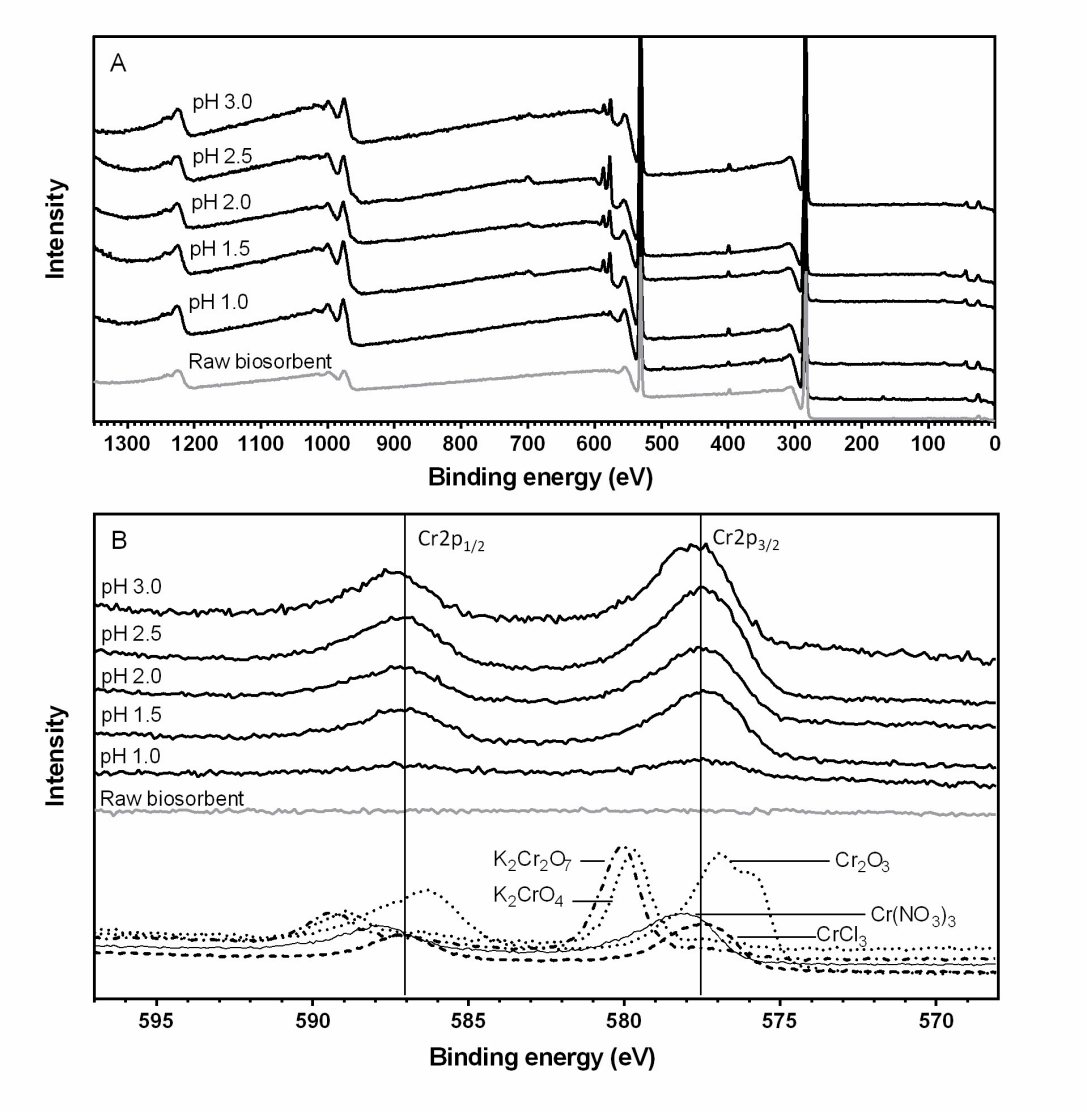
Wide-scan (A) and Cr 2p (B) XPS spectra of chromium-unloaded QCS (gray color line) and chromium-loaded QCS (black color line), and standard trivalent and hexavalent chromium compounds.

Fig 6 displays the wide-scan (Fig 6A) and the high-resolution (Fig 6B) XPS spectra collected from the Cr 2p core region of the raw (i.e., chromium-unloaded) and chromium-loaded QCS biomass obtained after chromium biosorption at the various influent pH values.

All the wide-scan XPS spectra reveal the existence of nitrogen, carbon, and oxygen on the biomass surface (Fig 6A). Likewise, the wide-scan (Fig 6A) and high-resolution (Fig 6B) XPS spectra of raw QCS indicate that chromium ions were not present on the biosorbent surface, whereas those of chromium-loaded QCS reveal important contributions of the metal bound to the biosorbent.

The XPS Cr 2p region spectra of the chromium-loaded QCS biomass obtained after chromium biosorption at influent pH values of 1.0, 1.5, 2.0, 2.5, and 3.0 show significant bands at binding energies of 577.5, 577.5, 577.6, 577.5, and 577.4 eV, respectively, which are assigned to Cr 2p_3/2_ orbitals, as well as bands at binding energies of 587.6, 587.2, 587.0, 587.1, and 587.7 eV, respectively, which are designated to Cr 2p_1/2_ orbitals. Cr 2p_3/2_ and Cr 2p_1/2_ orbitals for Cr(III) compounds are assigned at 577 eV and 587–587.5 eV, respectively [2, 39]. These binding energy values are very much alike to the ones observed in the XPS Cr 2p region spectra of chromium-loaded QCS.

Similarly, the XPS Cr 2p region spectra of Cr(III) [CrCl_3_·6H_2_O, Cr(NO_3_)_3_, and Cr_2_O_3_] and Cr(VI) [K_2_Cr_2_O_7_ and K_2_CrO_4_] reference compounds are displayed in Fig 6B. Signals of CrCl_3_·6H_2_O, Cr(NO_3_)_3_, and Cr_2_O_3_ (Cr(III) standards) appear at 577.6, 578, and 577 eV, respectively, which are assigned to Cr 2p_3/2_ orbitals, and at 587.3, 588.1, and 586.3 eV, respectively, which are designated to Cr 2p_1/2_ orbitals. These binding energies for the Cr 2p_1/2_ and Cr 2p_3/2_ orbitals are very much alike to the ones observed in the XPS spectra of metal-loaded QCS obtained after chromium biosorption at the various inlet solution pH levels for which assays were performed. It is apparent that the spectra of the chromium-loaded QCS biomass are well matched with those of Cr(III) species.

Contrastingly, signals of Cr(VI) compounds, K_2_Cr_2_O_7_ and K_2_CrO_4_, appear at binding energies of 580 and 579.7 eV, respectively, corresponding to Cr 2p_3/2_ orbitals, and at 589.4 and 588.9 eV, respectively, corresponding to Cr 2p_1/2_ orbitals. These binding energies for the Cr 2p_1/2_ and Cr 2p_3/2_ orbitals are higher than the ones observed in the XPS spectra of metal-loaded QCS. These results clearly show that there is no Cr(VI) associated with the surface of chromium-loaded QCS.

Therefore, the XPS spectra of chromium-loaded QCS biomass indicate the presence of Cr(III) but not of Cr(VI) on the QCS biomass. According to these results, it can clearly be concluded that most or all of the chromium bound to the QCS surface is in trivalent form.

**Fig 7:**
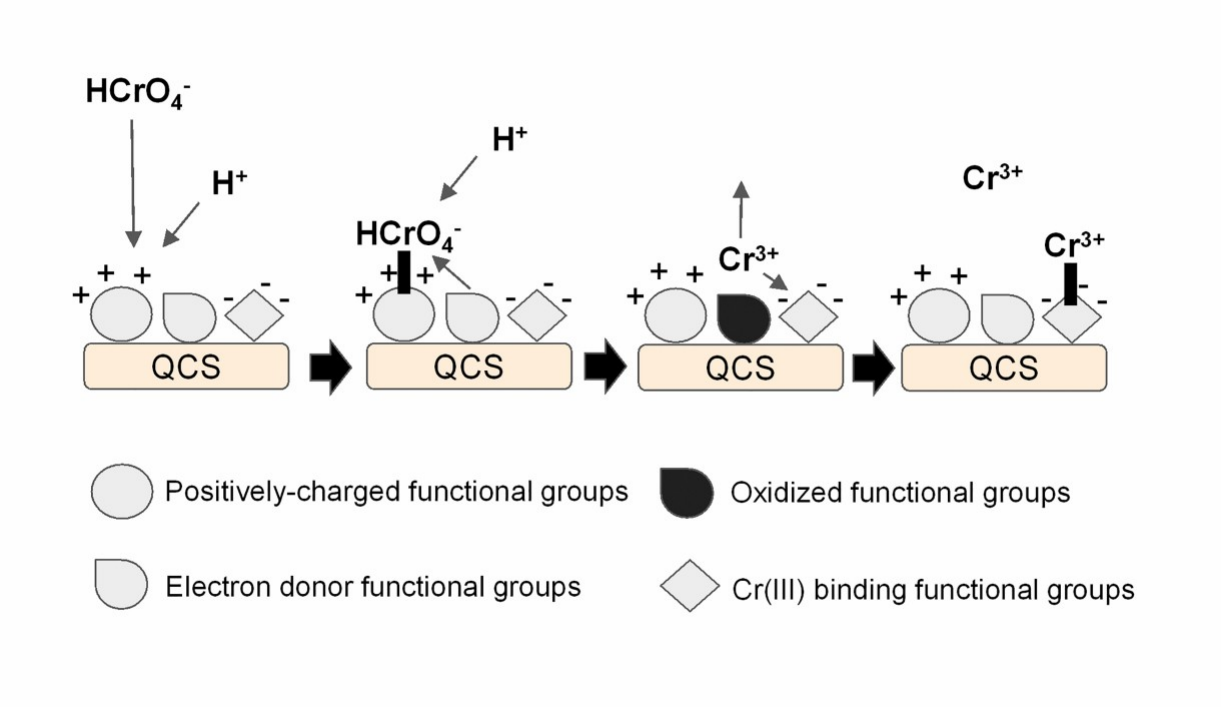
Proposed mechanism for Cr(VI) removal by QCS [40].

Kinetic and XPS results indicate that the mechanism for the removal of Cr(VI) from acidic aqueous solutions by QCS involves the binding of Cr(VI) oxyanions to positively charged groups, present at the QCS surface. Subsequently, the Cr(VI) species are reduced to Cr(III) by adjacent electron donor groups, and then the Cr(III) ions generated are partially bound to the QCS biomass and partially released into the liquid phase (Fig 7) [40]. Kinetic results also indicate that the amount of Cr(III) that is released into the aqueous solution and that bound to QCS is highly dependent on solution pH.

This mechanism has also been found for Cr(VI) removal from aqueous solution by lignocellulosic substrate [32], *Arthrobacter viscosus* [39], grape stalks and yohimbe bark [33], pine needle, pine bark, pine cone, banana skin, green tea waste, oak leaf, walnut shell, rice straw, peanut shell, sawdust, orange peel, rice husk [40], and Hass avocado shell [2].

## Conclusions

Acorn shell from *Quercus crassipes* Humb. & Bonpl. was found to be an effective biomaterial for continuous removal of both total chromium and Cr(VI) from aqueous solution, in a fixed-bed column. Results indicate that the breakthrough curves for the removal of total chromium and Cr(VI) are dependent on influent Cr(VI) solution pH, biosorbent bed height, influent flow rate, and influent Cr(VI) concentration. The highest total chromium biosorption capacity was 129.87 mg g^-1^, and this was attained at influent solution pH of 2.0, 3.5 cm bed height, 0.75 mL min^-1^ flow rate, and 400 mg L^-1^ influent Cr(VI) concentration. An inclusive description of the breakthrough curves for total chromium removal can be accomplished by applying the dose–response model. The mechanism of Cr(VI) removal from acidic aqueous solutions was found to involve the binding of Cr(VI) oxyanions to positively charged groups present at the QCS surface, followed by the reduction of Cr(VI) species to Cr(III) by adjacent electron donor groups. Subsequently, the generated Cr(III) ions become partially bound to the QCS biomass and are partially released into the liquid phase. Furthermore, there is a need for further mathematically modeled studies, concerning chromium desorption and QCS regeneration resulting from several consecutive biosorption-desorption cycles, in order to progress from chromium biosorption process technology at laboratory scale, through to pilot and then industrial scale.

## Abbreviations

AIC: Akaike information criterion
PZC: point of zero charge
QCS: shell of the acorn from *Quercus crassipes* Humb. & Bonpl.
RMSE: root mean squared error
SSE: sum of squared errors
XPS: X-ray photoelectron spectroscopy.
